# Influence of Drying Methods on the Post-Harvest Quality of Coffee: Effects on Physicochemical, Sensory, and Microbiological Composition

**DOI:** 10.3390/foods14091463

**Published:** 2025-04-23

**Authors:** Danilo José Machado de Abreu, Mário Sérgio Lorenço, Gilson Gustavo Lucinda Machado, Joana Moratto Silva, Estela Corrêa de Azevedo, Elisângela Elena Nunes Carvalho

**Affiliations:** 1 Microbiology Agricultural Sector, Natural Sciences Institute (ICN), Federal University of Lavras (UFLA), Lavras 37203-202, MG, Brazil; 2 Institute of Science, Technology and Innovation (ICTIN), Federal University of Lavras, São Sebastião do Paraíso 37950-000, MG, Brazil; mslorenco@gmail.com; 3 Food Science Sector, School of Agricultural Sciences of Lavras (ESAL), Federal University of Lavras (UFLA), Lavras 37203-202, MG, Brazil; gilson.machado1@estudante.ufla.br (G.G.L.M.); joanamttsilva@gmail.com (J.M.S.); estela.azevedo@estudante.ufla.br (E.C.d.A.)

**Keywords:** post-harvest technology, moisture, *Coffea arabica* L., Specialty Coffee Association, self-organizing maps, artificial neural networks

## Abstract

This study evaluated the impact of different drying methods on the physicochemical, microbiological, and sensory qualities of coffees produced in the Campos das Vertentes (CV) and Alta Mogiana (AM) regions of Brazil. The sun-drying (S), sun-drying combined with rotary mechanical dryer (SM), and CoffeeDryer^®^ mechanical dryer (C) methods were compared at different harvest times for the same crop (2024). The results indicated that CoffeeDryer^®^ preserved relatively high levels of phenolic compounds and antioxidant activity, reaching 3.24 g of gallic acid equivalents per 100 g (g EAG·100 g^−1^) and 47.96% antioxidant protection in the coffees produced in Alta Mogiana, whereas the sun-dried coffees presented relatively low values (2.20 g EAG·100 g^−1^ and 28.96% protection). In the Campos das Vertentes region, C maintained 2.78 g EAG·100 g^−1^ phenolic compounds and 50.29% antioxidant protection, outperforming combined drying (2.48 g EAG·100 g^−1^ and 41.17%). Regardless of the region and time of harvest, the coffees dried by C had a water activity of less than 0.6 and more stable moisture content (7.73–10.42%), reducing the possibility of proliferation of filamentous fungi and, consequently, mycotoxins. In the sensory evaluation, CoffeeDryer^®^ guaranteed higher scores for fragrance/aroma and flavor, allowing the coffees to reach 80 to 81 points on the SCA scale, which is classified as special. Thus, the use of CoffeeDryer^®^ proved to be an efficient alternative for optimizing coffee drying, preserving its chemical and microbiological qualities, and enhancing its commercial and sensory value.

## 1. Introduction

Coffee is one of the most consumed beverages in the world and one of the most traded commodities globally [1,2]. The largest coffee-producing countries are Brazil, Vietnam, and Colombia, whereas the European Union and the United States of America are the largest consumer and importer markets. Coffee is a growing market due in part to increased consumption in emerging economies and a stronger interest in specialty coffees and product innovations in developed countries [3,4].

Coffee quality is the result of the interaction of a number of factors, starting in the field and extending to the cup. The variety of beans grown, soil and climate conditions, and agricultural practices directly influence their characteristics [4]. Post-harvest processing, roasting, and grinding shape the sensory profile of the drink, while consumers’ personal preferences determine their perception of quality. This complexity makes coffee a unique drink, with infinite possibilities for combining flavors and aromas [5].

The coffee drying stage is crucial to the quality of the product and requires precise control of the environmental conditions [6,7]. Natural sun drying, a traditional and low-cost method, is influenced by climatic factors and has a variable drying time, which can last for days. This technique, although simple, exposes the grains to contaminants and adverse weather conditions, compromising the quality and uniformity of the batch. Given these limitations, mechanical drying techniques have emerged, offering greater control over the process and shorter drying times. Mechanical drying uses specific equipment and artificial heat sources, allowing the grains to dry quickly and evenly [8].

Drying coffee is a complex process that requires meticulous control of various parameters to guarantee the final quality of the product. Temperature, relative moisture, ventilation, and the surface area of beans are interdependent factors that directly influence drying efficiency and the sensory characteristics of coffee [7]. Sun drying on cement or brick patios, commonly known as terreiro drying, is one of the oldest and most widely used post-harvest methods for coffee processing. In this method, coffee cherries or parchment beans are spread in thin layers on flat surfaces and exposed to direct sunlight, with periodic manual or mechanical turning to ensure uniform drying. The process typically spans several days and highly depends on weather conditions such as solar radiation, ambient temperature, and relative humidity. While terrestrial drying is cost-effective and environmentally friendly due to its low energy input, it presents challenges related to drying uniformity, microbial contamination, and exposure to environmental contaminants. Variations in the drying rate across the bean mass can lead to heterogeneity in the moisture content, which may affect the cup quality and storage stability. Despite these limitations, proper management of drying time, turning frequency, and protection from rain and dust can result in high-quality coffee suitable for the specialty market [9,10,11].

Given this context, mechanical dryers can overcome the limitations of sun drying by offering greater control over process conditions and promoting more uniform drying of the coffee beans. Technologies such as CoffeeDryer^®^ exemplify this advanced mechanization by integrating precise temperature and airflow control. The drying process occurs in two stages: an initial static phase, during which the beans remain at rest and receive indirectly heated air until reaching approximately 30% moisture content; and a dynamic phase, characterized by cyclic movement of the beans that accelerates moisture removal. The system operates via cross, reverse, and countercurrent airflows distributed through alternating 7 cm columns of coffee and empty spaces, which facilitate the evacuation of humid air and optimize thermal performance. The heated air, generated by turbines and kept separate from combustion gases, maintains the bean mass temperature below 40 °C, preventing thermal damage. This configuration results in faster, more homogeneous, and more efficient drying, preserving the physical integrity of the beans and contributing to the final quality of the coffee [11,12].

Therefore, this study aims to compare the effects of three drying methods—traditional sun drying (S), combined sun drying and mechanical rotary drying (SM), and a high-efficiency mechanical dryer (CoffeeDryer^®^, C)—on the post-harvest quality of *Coffea arabica* beans. These methods were selected because they represent the most widely adopted technologies in Brazilian coffee-producing regions, ranging from artisanal to industrial scales. While previous studies have investigated the individual effects of sun or mechanical drying, there is a lack of integrated assessments comparing these three strategies under the same experimental conditions. Furthermore, coffee beans harvested during different periods of the same season (beginning, middle, and end) may exhibit natural variations in maturation and composition, which directly influence their physicochemical and sensory profiles. Thus, evaluating the performance of drying methods across multiple harvests offers a more comprehensive understanding of their consistency, robustness, and influence on coffee quality attributes.

## 2. Materials and Methods

### 2.1. Plant Material and Study Region

**Figure 1:**
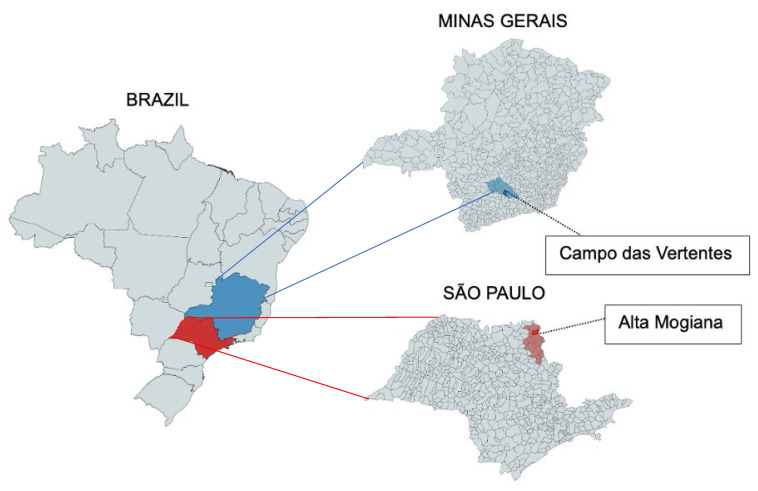
Coffee-producing regions of Brazil where green coffee beans were harvested (in red: Alta Mogiana region, São Paulo; in blue: Campo das Vertentes region, Minas Gerais).

The experiments were carried out in Brazil’s coffee-producing regions: (i) the Alta Mogiana region (20°23′50″ S and 47°25′13″ E, altitude 996 m) located in the state of São Paulo; (ii) the Campo das Vertentes region (21°12′46″ S and 44°35′54″, altitude 922 m) in the state of MG (Figure 1). The green coffee beans used in this work were *Coffea arabica* L. The experiments were carried out during the 2024 harvest.

### 2.2. Experimental Design for Drying Green Arabica Coffee Beans

The research was carried out through 2 experiments with a completely randomized design (DIC) in a factorial arrangement. The factors for the Campo das Vertentes region were as follows: 2 drying methods (sun dryer and CoffeeDryer^®^ (Dryeration, Cachoeirinha, Rio Grande do Sul, Brazil)) and 3 harvest time (beginning, middle, and end of harvest). The harvest was carried out in June/July 2024. The cherry coffee beans (“bica corrida” type) were harvested mechanically (average moisture 35%) and divided into two batches: batch 1 was taken straight to the CoffeeDryer^®^, where it was dried to approximately 11% moisture for 24 h; batch 2 was taken straight to the cement surface yard to undergo the traditional method of drying exposed to the sun, where it remained for 10 days (up to 11% moisture on average).

For the Alta Mogiana region, 2 drying methods were used (sun dryer with mechanical dryer and CoffeeDryer^®^ with a capacity of 40,000 L × 3 harvest times (beginning, middle, and end of harvest)). The harvest took place in August/September 2024. The coffee beans (“bica corrida” type) were harvested mechanically (average moisture 35%) and divided into 2 batches: batch 1 was taken straight to the CoffeeDryer^®^ dryer (Dryeration, Cachoeirinha, Rio Grande do Sul, Brazil), where it was dried to approximately 11% moisture (for 24 h); batch 2 was taken straight to the asphalt surface to undergo the traditional method of drying exposed to the sun, where it remained for 8 days; after this period, it was transferred to the rotary dryer (Pinhalense, Espirito Santo do Pinhal, São Paulo, Brazil) for 48 h (11% moisture on average). The CoffeeDryer^®^ technology used in the experiment is based on a drying system that combines crossflow air, periodic reversal, and countercurrent air. The flow of the equipment was reversed every ten minutes, ensuring that the hot air reached the beans alternately. Drying is carried out in narrow columns of grains approximately 7 cm thick.

All the experiments were carried out in triplicate. The factors are as follows: B: beginning of harvest; M: middle of harvest; E: end of harvest; CV: Campo das Vertentes; AM: Alta Mogiana; S: sun dryer; SM: sun + mechanical dryer; C: coffee dryer.

### 2.3. Physicochemical Analysis

A total of 100 randomly selected dried green coffee beans were measured to determine their size parameters. The following three main dimensions were measured for each seed via a digital caliper: length (L), width (W), and thickness (T). In addition, the mean geometric diameter (MGD), mean arithmetic diameter (MAD), sphericity (φ), circularity (C), surface area (S), expressed in mm^2^, volume (V), expressed in mm^3^, and unit mass and mass of 1000 beans, which were measured according to Mohsenin [13].

Proximal composition analyses (moisture, ash, protein, lipids, and total carbohydrates) and total titratable acidity (TTA) were conducted in triplicate. The results are expressed in g·100 g^−1^ (%). Moreover, analyses of hydrogen ionic potential (pH) (TEC5, Tecnal, Piracicaba, São Paulo, Brazil) and soluble solids were conducted via a digital refractometer (AR200, Reichert Analytical Instruments, Depew, NY, USA), and water activity (Aw) was determined via an Aqualab device (Aqualab CX-2, Decagon Devices, Washington, DC, USA) in five repetitions for all three batches of green coffee beans [14].

### 2.4. Colorimetric Parameters

Instrumental color parameters were determined via a colorimeter (Colorquest XE HunterLab, Reston, VA, USA). These results were expressed using the CIE system in terms of L*, chroma (C), and hue angle (H°). These analyses were conducted with all three batches of green coffee beans in 15 repetitions.

### 2.5. Phenolic Compounds and Antioxidant Capacity

For determination of the antioxidant capacity, a ternary extract was prepared with methanol 50% (*v*/*v*)/acetone 70% (*v*/*v*)/distilled water (2:2:1). Briefly, 20 mL of 50% methanol was added to 2.5 g of sample, which was left to stand for 1 h. The extract was filtered and transferred to a 50 mL flask. Then, 20 mL of 70% acetone was added, and the same procedure was performed. The filtrate was added to the same flask previously used so that the final volume was 50 mL with distilled water [15]. All extracts were placed in amber flasks and stored in a freezer at −18 °C until analysis.

Phenolic compounds were determined via the Folin-Ciocalteu method [16]. Determination was carried out at 720 nm in a spectrophotometer (Biochrom EZ Read 400, Cambridge, UK). The results are expressed in g of gallic acid equivalents per 100 g of green coffee beans [15].

The determination of β-carotene bleaching was conducted [15]. Determination was carried out at 470 nm in a spectrophotometer (Biochrom EZ Read 400, Cambridge, UK). The results are expressed as a percentage of protection (%). Antioxidant capacity analyses were carried out in triplicate [15].

### 2.6. Hygienic and Sanitary Qualities of Dried Coffee Beans

The procedure was performed on the dried coffee beans before husking. For the total count of molds and yeasts, dichloran rose bengal chloramphenicol base (DRBC) agar was utilized. The plates seeded on the surface were incubated at 28 ± 2 °C for 72 h [17]. All microorganism counts were expressed in colony-forming units per gram (log UFC.g^−1^).

### 2.7. Isolation and Morphological Identification of Fungi

Fungal isolation was conducted via surface plating on dichloran rose bengal chloramphenicol base (DRBC) agar, a medium that selectively supports filamentous fungi. For each treatment, 100 coffee beans were randomly selected, and groups of 10 beans were placed onto DRBC plates in triplicate. The plates were incubated at 25 °C for 7 days. Following the incubation period, the number of beans exhibiting visible fungal growth was recorded, and the percentage of contamination was calculated.

Fungal colonies were subsequently subcultured onto malt agar (MA) and incubated again at 25 °C for 7 days to obtain pure isolates. Morphological identification was performed via standardized mycological procedures. For isolates belonging to the genera Aspergillus and Penicillium, cultures were further cultivated on Czapek yeast agar (CYA) at both 25 °C and 37 °C, as well as on malt extract agar (MEA) at 25 °C. For all other genera, identification was carried out using MEA at 25 °C. Macroscopic characteristics (colony color, reverse color, colony diameter, exudate production, and sclerotia formation) and microscopic features (structure and size of conidiophores, metulae, phialides, and ornamentation of conidia) were assessed after incubation. The identification of isolates was carried out via standard taxonomic keys and mycological manuals [18,19,20,21].

### 2.8. Ochratoxin A Detection

Ochratoxin A (OTA) detection was performed using an immunoassay method (Nano Smart, Serra, Brazil). The immunoassay was carried out according to the manufacturer’s instructions. The detection limit of the kit is 2.5 µg/kg (ppb) for ochratoxins [6].

### 2.9. Sensory Analysis

The samples were selected with a sieve size of 16 or greater and were free of intrinsic and extrinsic defects. The roasting process followed the protocol established by the Specialty Coffee Association (SCA). The sensory evaluation was conducted with five cups per sample, which were analyzed by three calibrated Q-Graders following the same protocol. Seven sensory attributes were considered in the analysis, including fragrance/aroma, flavor, finish, acidity, body, balance, and overall evaluation, all of which were scored on a scale of 6 to 10. In addition, the attributes of uniformity, sweetness, and cleanliness of the cup were also evaluated [22].

### 2.10. Statistical Analysis

The results are expressed as the means ± standard deviations. Analysis of variance (ANOVA) was applied to the physicochemical and microbiological parameters, followed by a Scott–Knott test (*p *< 0.05) for the times the dried coffee beans were collected and a Student’s t-test for the different drying methods. A value of *p *< 0.05 was considered statistically significant. Multivariate statistical methods and Pearson’s correlations was carried out in R Studio (Version 2024.12.0+467 ). The self-organizing maps (SOMs) was conducted in Matlab R2020a (9.8.01323502) were applied SOM toolbox 2.1. 

## 3. Results and Discussion

### 3.1. Evaluation of the Physicochemical Parameters of SOMs

**Figure 2:**
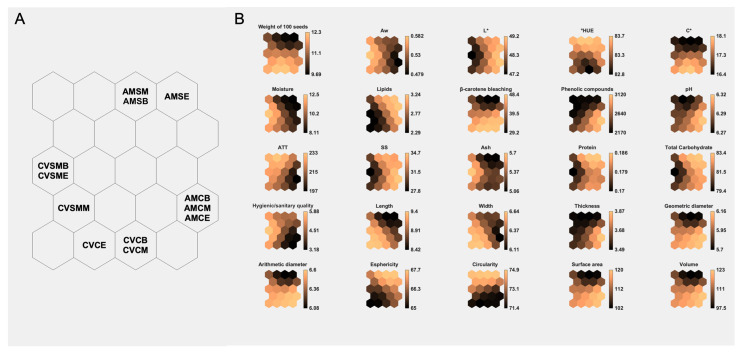
Artificial neural network evaluation of physicochemical and microbiological variables. (**A**) Distribution of the different drying methods and harvest times for green coffee beans (*Coffea arabica* L.) from the Campo das Vertentes and Alta Mogiana mesoregions in the SOM. (**B**) SOM components for 25 input variables. The colors indicate the component’s value in the weight vector of each unit on the map, according to the colored bars on the right-hand side.

Self-organizing maps (SOMs) show how the treatments are distributed among the neurons of the artificial neural network (ANN). In these maps, when the treatments share the same neuron, represented by the hexagon, they are highly similar, whereas the completely different samples are far apart and in other hexagons. Therefore, the physicochemical and microbiological parameters of the coffees from the studied mesoregions differ. The coffee from the Campo das Vertentes mesoregion is at the bottom left, and the coffee from the Alta Mogiana mesoregion is at the bottom right and top of the distribution of self-organizing maps (Figure 2A).

For coffees produced in the Campo das Vertentes and Alta Mogiana mesoregions, there was a significant difference (*p *> 0.05) among the different drying methods evaluated as well as among the harvest times evaluated in this study, depending on the parameter evaluated (Table S1). Because they are in different neurons, the coffees dried using the solar method combined with the rotary mechanical dryer differ from the coffees dried via the CoffeeDryer. The average soluble solids values ranged from 26.66 to 33.66% for the beans produced in the CV region and from 31.66 to 36.67% for the coffee beans from the AM region. It was therefore possible to observe that the coffee beans dried with the CoffeeDryer had a relatively high soluble solids content, approximately 19.61% for CV and 13.66% for AM. The soluble solids present in coffee, which are composed of acids, sugars, precursors of aromatic compounds, and other substances, play crucial roles in determining the quality of the drink. Specifically, they are mainly responsible for the sensation of the body, which refers to the sensory perception of density and a more pleasant texture in the coffee drink [23,24,25]. The use of the CoffeeDryer made it possible to maintain the characteristics related to the maturation of the coffee due to the control and stability of the temperature applied during drying [26].

The drying method directly influences the final moisture content of the coffee beans. The ideal moisture content for marketing and exporting coffee beans has a maximum tolerance of 12.5% (*w*/*w*), which means that the coffees evaluated, regardless of the drying method, are in accordance with normative instruction No. 08/2003 [27]. Except for the coffee dried with SM at the beginning of harvest in the Campo das Vertentes mesoregion, which presented a moisture value of 14.07%, this value is expected for sun-dried coffees [9]. Despite this, the C-dried green coffee beans in Campo das Vertentes had a moisture content of 9.91–10.42%, whereas those in Alta Mogiana had a lower moisture content, ranging from 7.73 to 9.01%, and were more efficient in terms of removing water content, thus meeting international marketing standards [11].

Water activity is another parameter that was evaluated and is related to the preservation and stability of green coffee beans. This parameter refers to the fraction of free water available in the bean that microorganisms can use for growth. Microorganisms develop in food matrices when water activity values are above 0.5, especially filamentous fungi [28]. All the treatments evaluated had water activity values less than 0.6, guaranteeing the grains’ preservation during storage and marketing and preventing the proliferation of fungi and microbiological degradation [29]. Notably, the C-dried coffees had lower water activity values (*p *< 0.05) than the other drying methods.

The physical characteristics of the green coffee beans dried via the different methods were significantly different in terms of weight and colorimetric parameters, such as luminosity and chroma (*p *< 0.05) (Figure 2B). There was a significant difference in the mass of one hundred green coffee beans between harvest times. For the coffee beans produced in the CV, the dry coffees harvested in the middle of the harvest and using the SM had the highest weight (11.77 g), whereas the coffees harvested at the beginning and end of the harvest weighed 11.21 g. The green coffee beans harvested during the harvest in the Alta Mogiana region weighed less than those from the Campos das Vertentes region. In particular, the average weight of the coffee beans subjected to sun drying was 9.62 g. These variations are associated with abiotic factors such as altitude, irrigation regime, cultivation temperature, and genetic differences between cultivars [30]. A significant difference was also observed between the drying methods (*p *< 0.05). Compared with the CoffeeDryer^®^ method, the mass loss of green coffee beans dried via the sun or combined methods was greater, with reductions of approximately 15% in Alta Mogiana and 10% in Campos das Vertentes. The CoffeeDryer^®^ technology was more effective in minimizing weight loss during drying, which is advantageous for maintaining bean quality and maximizing commercial value due to the preservation of bean mass [22,31].

In both of the evaluated regions, coffee beans dried via the CoffeeDryer^®^ (C) method presented a significantly greater volume than those dried via sun drying (S) or the combined sun and mechanical method (SM) (*p *< 0.05; Figure 2B). Variations in volume and mass are relevant parameters for post-harvest management as they influence storage capacity and transport logistics and can assist in determining optimal packaging solutions and seed distribution strategies [32].

A positive relationship was observed between moisture content and the circularity of coffee beans, with higher moisture content tending to exhibit greater circularity values. This trend was particularly evident in sun-dried samples from the Alta Mogiana region, especially at the beginning (75.12% circularity; 7.73% moisture) and end of the harvest (75.62% circularity; 8.86% moisture). However, unlike what was presented by Araujo et al. [33], the data indicate that roundness did not increase with reductions in moisture. Contrarily, the opposite was the case: wetter coffee beans tended to maintain greater roundness. This type of variation directly influences the size of the green coffee beans such that the beans from Alta Mogiana are shorter (beginning: 8.49 mm; middle: 8.42 mm; end: 8.29 mm) than the beans from Campo das Vertentes, where the length values are greater. The beans dried via the sun method in Alta Mogiana were shorter, showing that the time of harvest and the drying method influenced the morphology of the beans. For sphericity, more spherical beans tend to have greater circularity, reinforcing the idea that geometric changes directly influence the classification of coffees and their commercial acceptance.

Although drying improves the stability of food, it can induce changes in chemical and physical reactions, such as deterioration in color or a darkening of the beans. The SM-dried coffee was a darker bean, i.e., it had a lower luminosity (L* value) (46.90) than the C-dried coffee (49.20). This significant difference in luminosity was detected only in the dried coffee beans collected at the beginning of the CV harvest, where the luminosity value decreased by 4.67%. For AM, a similar behavior was observed at different harvest times in the beans harvested at the beginning (SD: 49.19; C: 49.93) and at the end of the harvest (SD: 48.10; C: 49.67) (Figure 2B). Therefore, the drying method in the yard shows that the variable temperature and long duration of coffee bean exposure influenced the process of degrading pigments sensitive to heat, light, and oxygen, reducing the color of the beans, making them darker (lower L* value) and less bright [34]. The L* value ranges from dark (0) to light (100) and indicates the lightness or darkness of the coffee beans. Thus, higher luminosity values suggest less thermal degradation or darkening [35]. With these variations, the chroma parameter was also influenced, following the same behavior as that observed for luminosity, with an average difference of 1.02 between the drying methods. In addition, the acceptance of grain by the market may be impaired since this behavior may continue during storage. Given this, the C-dried method is more efficient as it has no negative effect on colorimetric parameters due to its high heat transfer efficiency and drying speed [36] of within 24 h.

### 3.2. Microbiological Analysis by SOM, Presence/Absence of Ochratoxin A, and Morphological Identification of Filamentous Fungi

**Figure 3:**
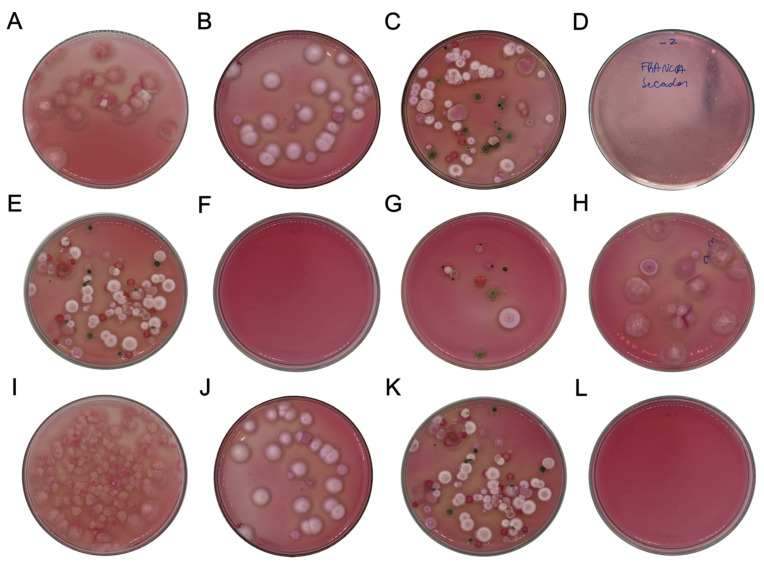
Hygienic and sanitary evaluation of *Coffea arabica* L. dried via different methods ((**A**) CVSMB; (**B**) CVCB; (**C**) AMSB; (**D**) AMCB; (**E**) CVSMM; (**F**) CVSMM; (**G**) AMSM; (**H**) AMCM; (**I**) CVSME; (**J**) CVSME; (**K**) AMSE; (**L**) AMCE). B: beginning of harvest; M: middle of harvest; E: end of Harvest; CV: Campo das Vertentes; AM: Alta Mogiana; S: sun dryer; SM: sun + mechanical dryer; C: CoffeeDryer).

The population of filamentous fungi varied significantly according to harvest time in both regions evaluated. Additionally, the drying method had a significant effect on the fungal contamination level (*p *< 0.05), as shown in Figure 3. Greater contamination was observed in samples from the CV and AM regions when the sun drying (S) and combined (SM) methods were used, suggesting an increased risk of microbial spoilage during storage under these conditions. The variation in the population across the regions was influenced by the time of harvest (CV: B: 6.47 log CFU.g^−1^; M: 7.35 log CFU.g^−1^; E: 5.10 log CFU.g^−1^; AM: B: 5.02 log CFU.g^−1^; M: 3.99 log CFU.g^−1^; E: 4.87 log CFU.g^−1^). However, when drying was carried out with the CoffeeDryer, there was an average reduction in the fungal population of 38% and 44% for the AM and CV regions, respectively (Tables S1 and S2: Supplementary Material). Thus, the highest microbial population was observed in grains with the highest moisture and water activity, suggesting that faster and more controlled drying of C reduces the proliferation of microorganisms.

Due to the presence of fungal populations in green coffee beans, a morphological identification of the species was carried out, and the species identified are shown in Table 1.

Coffee quality is influenced by factors such as the choice of *Coffea arabica* L. variety, climatic conditions during cultivation, processing method, and, especially, storage. Drying is a critical stage as it directly impacts the stability of beans during storage. Colonization by toxigenic fungi represents a potential risk of contamination by mycotoxins and is therefore a relevant factor in assessing the quality of coffee beans [36,37].

In this study, five fungal genera were identified in the beans analyzed and were influenced by the drying method used (Table 1). The fungi found in C-dried grains are not widely recognized as mycotoxin producers, suggesting a lower toxigenic risk. In addition, the possible suppression of *Aspergillus* sp. over *Cladosporium* sp. was observed, a phenomenon relevant to fermentation and storage processes. The fungal diversity identified in this study corroborates the findings of Silva et al. [38] and Lu et al. [39] during the drying stage, highlighting the predominance of species commonly found in storage environments (Figure 3).

These data can be correlated with the presence of toxins such as ochratoxin A. In this study, regardless of the producing region, drying method, and harvest time, the detected value was less than 2.5 μg.kg^−1^ (Table 2). Thus, the green coffee beans under study comply with Brazilian and European Union legislation, where the limits for roasted or ground coffee beans are 10 μg.kg^−1^ and 5 μg.kg^−1^, respectively.

Although several species of toxin-producing fungi were found during coffee processing, the concentration of ochratoxin A is below the limit allowed by legislation. Therefore, coffee beans are suitable for commercialization without problems related to toxins as they will have undergone the roasting process, which is a necessary unit operation for the consumption of coffee beverages and also guarantees the elimination or reduction of these toxins [42]. 

### 3.3. Bioactive Compounds Produced by SOM

The preservation of bioactive compounds in coffee beans is directly related to the chemical and sensory qualities of the product. In this study, we evaluated the influence of drying methods on the levels of phenolic compounds, β-carotene antioxidant protection, and lipids in the Alta Mogiana and Campo das Vertentes regions (Figure 2B). In addition, the correlations between these compounds and factors such as moisture and water activity (Aw), which are fundamental for grain stability during storage, were analyzed.

In the Alta Mogiana region, the CoffeeDryer^®^ method preserved significantly (*p *< 0.05) more phenolic compounds (3.24 g EAG·100 g^−1^) than the sun dryer method (2.20 g EAG·100 g^−1^). In addition, the antioxidant protection of β-carotene was 65.5% greater in the CoffeeDryer^®^ sample (47.96% protection), indicating a positive effect on the retention of antioxidants essential for the chemical quality of the beans. On the other hand, lipids varied less significantly, with average values of 3.28% using the CoffeeDryer^®^ and 3.19% using sun drying.

In the Campo das Vertentes region, the CoffeeDryer^®^ also performed better in preserving bioactive compounds, although with a less marked difference. The average content of phenolic compounds was 2.78 g EAG·100 g^−1^ in the CoffeeDryer^®^ sample, whereas it was 2.48 g EAG·100 g^−1^ in the SM sample, resulting in 12% greater retention (*p *< 0.05). The antioxidant protection of β-carotene was also greater in the CoffeeDryer treatment (50.29% protection) than in the SM treatment (41.17%), indicating a 22% greater antioxidant capacity. With respect to lipids, preservation was 37.3% greater in the CoffeeDryer^®^ treatment (3.27%) than in the SM treatment (2.39%).

These findings indicate that, compared with traditional drying methods, the CoffeeDryer^®^ provides better preservation of bioactive compounds, ensuring greater antioxidant and sensory stability. The reduction in moisture and water activity during this process favors the retention of phenolic compounds, antioxidant capacity via the β-carotene method, and lipids, contributing to the improvement in the chemical and sensory qualities of the coffee. In addition, enzymatic oxidation, thermal degradation, and lipid volatilization are minimized in beans dried using the CoffeeDryer^®^, favoring product quality. These findings reinforce the importance of using controlled drying techniques to maximize the bioactive stability of coffee [36,37].

### 3.4. Scanning Electron Microscopy (SEM)

When coffee undergoes processing, including drying and roasting at high temperatures, it undergoes a process of volumetric expansion characterized by the loss of mass and the formation of large pores. These structural changes facilitate the migration of oils to the surface of the coffee bean matrix and promote the release of CO_2_. In addition, these changes can influence the stability of the grain during storage, increasing its susceptibility to oxidation and possibly adverse reactions, such as the development of rust [34]. 

**Figure 4:**
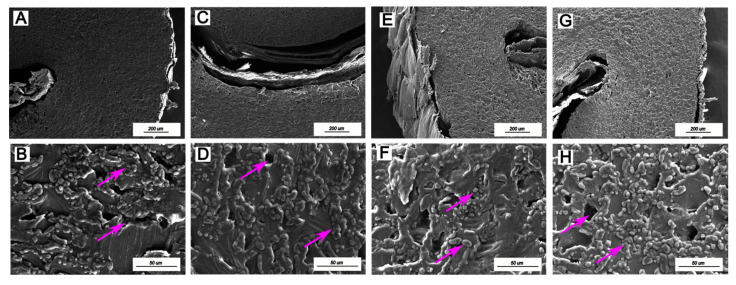
Scanning electron micrographs of coffee beans dried via different methods ((**A**,**B**): AMC; (**C**,**D**): AMS; (**E**,**F**): CVC; (**G**,**H**): CVSM)—CV: Campo das Vertentes; AM: Alta Mogiana; S: sun dryer; SM: sun + mechanical dryer; C: CoffeeDryer). The purple arrows indicate the structural changes observed in coffee beans dried by different drying methods.

The results revealed that the CoffeeDryer method resulted in a more preserved and homogeneous cell structure in the beans from both regions (AMC and CVC) (Figure 4A,B,E,F). The images revealed a compact, less fragmented surface, with fewer cracks and less degradation of the cellular matrix. In addition, the presence of globular particles on the surface of the beans was observed, which was possibly associated with the preservation of lipids and bioactive compounds, which are essential for the chemical and sensory stability of coffee.

In contrast, the grains subjected to sun drying (AMS and CVSM) presented more damaged microstructures, characterized by cracks and increased porosity, indicative of greater thermal and mechanical stress (Figure 4C,D,G,H). The presence of these cracks may be associated with greater exposure to oxygen, which favors the oxidation of bioactive compounds and the degradation of aromatic volatiles. This effect was more evident in the coffee beans produced in the Campo das Vertentes region, where SM drying resulted in greater fragmentation and structural disorganization, suggesting that this method may induce more internal tension in the beans due to variations in temperature and moisture during the drying process.

The formation of cracks and structural irregularities observed in CVSM suggests a negative impact on coffee quality, since these alterations can influence the ability of coffee beans to retain volatile compounds in their matrix, which are essential for the sensory profile of the product. In addition, structural degradation can promote the entry of oxygen and moisture, increasing the susceptibility of beans to microbiological deterioration and lipid rancidity [43].

### 3.5. Pearson’s Correlation

**Figure 5:**
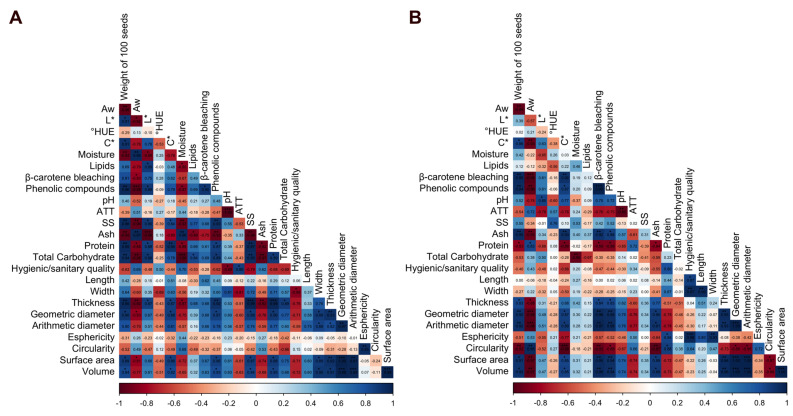
Pearson’s correlation of the physicochemical and microbiological variables of green coffee beans dried via different methods. ((**A**) Campo das Vertentes; (**B**) Alta Mogiana. (*) Correlation is significant at the 0.05 level; (**) correlation is significant at the 0.01 level; (***) correlation is significant at the 0.001 level.)

The quality of coffee beans is influenced by various physicochemical factors. Moisture is one of the parameters that most affects the preservation of bioactive compounds, lipid stability, and microbiological safety. The Pearson correlation analysis carried out for the Campo das Vertentes and Alto Mogiana regions revealed statistically significant relationships between these variables, providing fundamental information for understanding the impact of drying on bean stability (Figure 5).

For the Campo das Vertentes region, moisture was significantly correlated at different levels (*p *< 0.05, *p *< 0.01, and *p *< 0.001). A strong positive correlation was observed between moisture and water activity (0.95, *p *< 0.01), confirming that wetter grains have greater availability of free water, favoring biochemical reactions that can accelerate chemical and microbiological deterioration. In addition, there was a negative correlation between moisture and phenolic compounds (−0.87, *p *< 0.05), indicating that grains with greater moisture content are more prone to oxidation and the degradation of these compounds, which reduces their chemical stability and ability to protect against free radicals (Figure 5A). This result reinforces the need for the strict control of drying, since the reduction in moisture favors the retention of these essential compounds for the chemical quality of coffee.

In the Alto Mogiana region, similar patterns were observed (Figure 5B). The negative correlation between water activity and phenolic compounds was found to be strong (−0.96, *p *< 0.01), and the antioxidant capacity determined via the β-carotene bleaching method was −0.99, *p *< 0.001, confirming that the retention of bioactive compounds is directly associated with the control of moisture in the grains and is able to directly influence their biochemical stability and the development of enzymatic reactions throughout processing. The chemical quality of the beans was positively correlated with surface area (0.95, *p* < 0.01), thickness (0.95, *p *< 0.01), geometry (0.95, *p *< 0.01), and arithmetic diameter (0.95, *p *< 0.01), and the antioxidant capacity determined via the B-carotene bleaching method was greater, indicating that coffee beans with a larger exposed area are more prone to oxidation. This finding reinforces the hypothesis that controlling bean morphology during drying can play a significant role in preserving antioxidant compounds.

Therefore, moisture is a critical factor in preserving the quality of coffee beans as it directly affects the retention of bioactive compounds and their antioxidant and microbiological stability. These results reinforce the need for drying methods that guarantee an adequate balance between removing moisture and preserving the physicochemical characteristics of the beans, ensuring greater stability during storage and processing.

### 3.6. Sensory Analysis of the Coffee Beverages

The quality of the coffee drink was assessed via a sensory analysis carried out by Q-Graders, which allowed the effects of the drying methods to be compared. The different coffee drying methods used influenced the quality of the coffee drink (*p *< 0.05). This variation in sensory quality is caused by a change in the chemical composition of the coffee beans, which is highly influenced by microorganisms, enzymatic reactions, and environmental parameters such as temperature, presence/absence of oxygen, and processing time [10]. For the CV region, no differences were observed in the attributes of aftertaste, balance, and overall (*p *< 0.05). For the AM region, the same behavior was observed for acidity, body, aftertaste, balance, and overall. In addition, all the treatments received a score of 10 for uniformity, clean cup, and sweetness. The scores awarded to the coffees can be seen in Figure 5 and in the Supplementary Material (Tables S3 and S4).

With respect to the fragrance/aroma attributes, the CoffeeDryer method stood out in both regions, providing more intense sensory notes than those obtained with conventional methods. In Campos das Vertentes, the CoffeeDryer coffees scored higher throughout the harvest (B: 7.50; M: 7.33; and E: 7.16), whereas those subjected to combined drying (sun + mechanical) remained stable at 7.00. In the Alta Mogiana region, the CoffeeDryer samples also presented slightly higher values at the end of the harvest (7.50), whereas the S-drying values varied between 7.00 and 7.16. These findings suggest that the CoffeeDryer method contributes to the preservation and intensification of the volatile compounds responsible for the aroma, resulting in a more pleasant sensory experience.

The Specialty Coffee Association (SCA) established a standardized system for the sensory evaluation of coffees, using a scale of 0–100 points to classify the quality of the beans. When coffees have a final score of less than 80, the coffee is considered a commercial coffee and has less sensory complexity. Coffees with a final score between 80 and 84.99 are considered very good or specialty coffees; from 85 to 89.99, the coffee is classified as excellent. In addition, when a coffee has a total score of over 90, it is considered to be exceptional [44].

**Figure 6:**
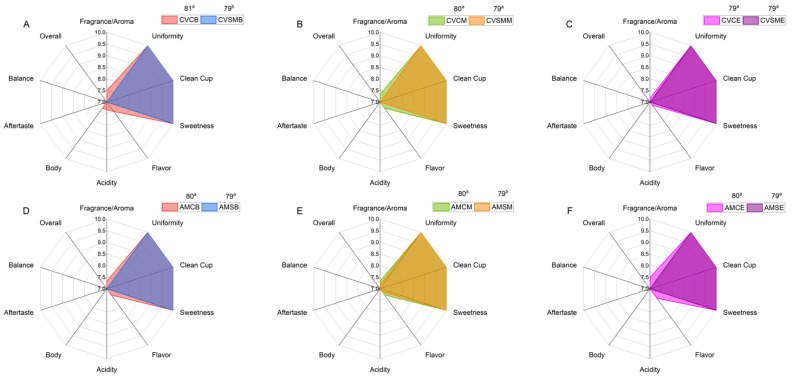
Sensory evaluation via the SCA cupping protocol of the mean scores of the attributes of coffee beverages from green coffee beans subjected to different drying methods during different harvest periods. (**A**,**D**) Beginning of harvest (B); (**B**,**E**) middle of harvest (M); (**C**,**F**) end of harvest (E) (CV: Campo das Vertentes; AM: Alta Mogiana; S: sun dryer; SM: sun + mechanical dryer; C: CoffeeDryer^®^). The means followed by lowercase letters for the final score are significantly different according to the Scott–Knott test (*p *< 0.05).

As a result, in the Campo das Vertentes region, only the coffees subjected to the CoffeeDryer^®^ surpassed the 80-point barrier and were classified as specialty coffees, especially those processed at the beginning (81) (Figure 6A) and middle (80) (Figure 6B) of the harvest. However, the coffee beans harvested at the end of the season, even when dried via the CoffeeDryer^®^ method, did not score differently from the coffees dried via the SM method, which scored below 80 points and were classified as commercial coffees (Figure 6C). For the Alto Mogiana region, the effects of drying followed the same behavior observed in CV. The CoffeeDryer^®^ maintained its advantage over traditional methods, and the coffee beans dried via this technology had final scores of 80, classifying them as specialty coffees, regardless of the time the beans were harvested and processed, suggesting that the aromatic compounds and the uniformity of the beans were preserved more efficiently. The coffees dried via the S method were classified as commercial coffees (Figure 6D–F), since moisture below 9%, as occurs in these coffees, leads to a risk of loss of aromatic compounds and excessive dryness, which can affect the flavor when roasted [7].

These differences are due to factors linked to pre- and post-harvest conditions, such as climatic changes, soil properties, grain handling, the chemicals used, efficient temperature control, and the dryer technology used [45]. Strictly controlling the temperature of the coffee beans while drying, reducing the movement of the beans to the minimum necessary level, and optimizing the airflow in contact with the coffee layer in convective drying, regardless of the equipment used, are process variables directly related to improving the sensory quality of the beverage [35,46].

## 4. Conclusions

The results of this study show that the drying method has a significant effect on the physical, chemical, microbiological, and sensory qualities of coffee. CoffeeDryer^®^ stands out as the most efficient alternative, guaranteeing the greater preservation of bioactive compounds, adequate control of moisture, and reduction in the microbiological load, minimizing the risk of contamination by fungi and mycotoxins. In addition, this method favored the retention of soluble solids, improving the flavor and aromatic complexity of the beverage.

In the sensory analysis, the coffees dried via CoffeeDryer^®^ presented higher scores for fragrance/aroma and flavor, maintaining greater stability in quality throughout the harvest. In the Campo das Vertentes region, this method allows the coffees to achieve scores classified as special; in Alta Mogiana, it ensures consistency in quality regardless of the time of harvest. Thus, the adoption of CoffeeDryer^®^ represents a step forward for the coffee industry, promoting a greater appreciation of coffee in the market and ensuring a high-quality product that is microbiologically safe.

## Figures and Tables

**Table 1:** Morphological identification of fungal genera/species present in green coffee beans subjected to different drying methods.

Region	Drying Method	Genus/Species
Alta Mogiana	CoffeeDryer^®^	*Fusarium* sp. *Penicillium implicatum*
Alta Mogiana	Sun Dryer	*Penicillium* sp. *Fusarium* sp.
Campo das Vertentes	CoffeeDryer^®^	*Rhizopus* sp. *Penicillium citrinum* *Cladosporium cladosporioides*
Campo das Vertentes	Sun + Mechanical dryer	*Aspergillus ochraceus* *Penicillium brevicompactum*

**Table 2:** Evaluation of the concentration of ochratoxin A in green coffee beans (*Coffea arabica* L.) from the Campo das Vertentes and Alta Mogiana mesoregions subjected to different drying methods.

Parameter	Campo das Vertentes			Alta Mogiana			Detection Limit (μg.kg^−1^)	
	Begin	Middle	End	Begin	Middle	End	Brazil ^1^	European Union ^2^
Ochratoxin A (μg.kg^−1^)	<2.5	<2.5	<2.5	<2.5	<2.5	<2.5	≤10.0	≤5.0

^1^ Refers to roasted coffee (ground or beans) and soluble coffee [40]. ^2^ Refers to roasted coffee beans and ground coffee [41].

## Data Availability

The original contributions presented in the study are included in the article; further inquiries can be directed to the corresponding author.

## Supplementary Materials

The following supporting information can be downloaded at: https://www.mdpi.com/article/10.3390/foods14091463/s1, Table S1. Physicochemical and microbiological analysis of Coffea arabica grains from the Campo das Vertentes region subjected to different drying methods during different harvest periods; Table S2. Physicochemical and microbiological analysis of Coffea arabica grains from the Alta Mogiana region subjected to different drying methods during different harvest periods; Table S3. Sensory evaluation of coffee beverages from genetic varieties of arabica coffee from Campo das Vertentes subjected to different drying methods; Table S4. Sensory evaluation of coffee beverages from genetic varieties of arabica coffee from Alta Mogiana subjected to different drying methods.
