# Supplementary material for: Influence of Drying Methods on the Post-Harvest Quality of Coffee: Effects on Physicochemical, Sensory, and Microbiological Composition
Source: Foods. 2025 Apr 23;14(9):1463. doi: 10.3390/foods14091463 (PMC12071231; doi:10.3390/foods14091463)
Supplement: Supplementary file 1 [file foods-14-01463-s001.zip › foods-3600802-supplementary.pdf]

foods

Influence of Drying Methods on the Post-Harvest Quality of Coffee: Effects on Physicochemical, Sensory, and Microbiological Composition

Danilo José Machado de Abreu [2], Mário Sérgio Lorenço [3], Gilson Gustavo Lucinda Machado [1], Joana Moratto Silva [1], Estela Corrêa de Azevedo [1] and Elisângela Elena Nunes Carvalho [1].

[1] Food Science Sector, School of Agricultural Sciences of Lavras (ESAL), Federal University of Lavras (UFLA), Lavras, Minas Gerais, Brazil;
[2] Microbiology Agricultural Sector, Natural Sciences Institute (ICN), Federal University of Lavras (UFLA), Lavras, Minas Gerais, Brazil;
[3] Forest Science, School of Agricultural Sciences of Lavras (ESAL), Federal University of Lavras (UFLA), Lavras, Minas Gerais, Brazil;
* Correspondent Author: Tel: +05562999948242, E-mail: danilo.mabreu@gmail.com

Table S1. Physico-chemical and microbiological analysis of Coffea arabica grains from the Campo das vertentes region subjected to different drying methods during different harvest periods[1].

Parameters	Campo Das Vertentes					
	Solar + Mechanical Dryer			Coffeedryer		
	Begin	Middle	End	Begin	Middle	End
Weight of 100 green coffee beans(g)	11.19±0.15^{bB}	11.77±0.31^{bA}	11.21±0.04^{bB}	12.76±0.11^{aA}	12.68±0.10^{bA}	12.51±0.09^{aA}
Aw	0.59±0.00 ^{aA}	0.58±0.00 ^{aA}	0.58±0.00 ^{aA}	0.53± 0.00^{bA}	0.53±0.00 ^{bA}	0.53±0.00 ^{bA}
L*	46.90±0.37^{bA}	47.14±1.70^{aA}	47.16±1.26^{aA}	49.20±0.044^{aA}	48.16±0.74^{aA}	48.25±0.56^{aA}
ºHUE	82.70±0.92^{aA}	81.89±1.14 ^{aB}	83.56±0.39^{aA}	83.70±0.22^{aB}	80.49±1.12 ^{aC}	85.04±0.79^{aA}
C*	16.86±0.54 ^{bB}	18.04±0.60 ^{aA}	16.57±0.68 ^{bB}	18.67±0.21 ^{aA}	18.49±0.16 ^{aA}	17.84±0.48 ^{aA}
Moisture(%)	14.07±0.16 ^{aA}	12.48±0.30 ^{aB}	12.10±0.30 ^{aB}	9.91±0.35 ^{bA}	9.94±0.20 ^{bA}	10.42±0.41 ^{bA}
Lipids (%)	2.18±0.08 ^{aA}	2.18±0.16 ^{aA}	2.36±0.10 ^{aA}	2.52±0.31 ^{aA}	2.38±0.30 ^{aA}	2.37±0.14 ^{aA}
β-Carotene bleaching (%protection)	42.62±4.44 ^{aA}	42.52±3.71 ^{aA}	38.37±0.46 ^{bA}	49.87±5.18 ^{aA}	46.28±1.39 ^{aA}	54.71±9.37 ^{aA}
Phenolics compounds (g EAG. 100g^{-1})	2.79 ±0.05 ^{bA}	2.21±0.14 ^{bA}	2.07±0.06^{bA}	2.79±0.04 ^{aA}	2.75±0.07 ^{aA}	2.81±0.17 ^{aA}
pH	6.28±0.01 ^{aA}	6.27±0.01 ^{bA}	6.28±0.01 ^{aA}	6.27±0.01 ^{aC}	6.32±0.02^{aA}	6.30±0.00 ^{aB}
ATT (%)	223.33±11.55 ^{aA}	236.66±5.77 ^{aA}	210.00±11.55 ^{aA}	233.33±5.77 ^{aA}	190.00±10.00 ^{bC}	223.33±0.00 ^{aB}
SS (%)	26.66±0.58 ^{bA}	27.66±0.58 ^{bA}	27.33±0.58 ^{bA}	33.00±1.00 ^{aA}	33.66±1.53 ^{aA}	31.00±1.00 ^{aC}
Ash (%)	5.88±0.14 ^{aA}	5.73±0.17 ^{bA}	5.70±0.16 ^{aA}	4.97±0.11 ^{bA}	5.19±0.06 ^{bA}	5.24±0.04 ^{bA}
Protein (%)	0.17±0.01 ^{aA}	0.19±0.01 ^{bA}	0.16±0.01 ^{aA}	0.19±0.01 ^{aA}	0.17±0.01 ^{aA}	0.18±0.01 ^{aA}
Total Carbohydrate (%)	77.69±0.14 ^{bB}	79.42±0.02 ^{aA}	79.66±0.44 ^{bA}	82.40±0.37 ^{aA}	82.29±0.05 ^{aA}	81.78±0.46 ^{aB}
Hygienic-sanitary quality (log. UFC.g^{-1})	6.47± ^{aB}	7.35± ^{aA}	5.10± ^{aC}	4,60± ^{aA}	0.00± ^{bB}	4.60± ^{aA}
Length (mm)	9.40±0.00 ^{aB}	9.44±0.00 ^{aA}	9.19±0.00 ^{bC}	9.35±0.00 ^{bC}	9.39±0.00 ^{bB}	9.46±0.00^{aA}
Width (mm)	6.60±0.00 ^{bB}	6.62±0.00 ^{bA}	6.56±0.00 ^{bC}	6.63±0.00 ^{aC}	6.81±0.00 ^{aA}	6.62±0.00 ^{aB}
Thickness (mm)	3.53±0.00 ^{bB}	3.55± 0.00^{bA}	3.51± 0.00^{bC}	3.65± 0.00^{aB}	3.67± 0.00^{bA}	3.61± 0.00^{aC}
Geometric diameter (mm)	6.01± 0.00^{bB}	6.05± 0.00^{bA}	5.96±0.00 ^{bC}	6.09±0.00 ^{aB}	6.16± 0.00^{aA}	6.09±0.00 ^{aB}
Arithmetic diameter (mm)	6.51± 0.00^{bB}	6.54±0.00 ^{bA}	6.42± 0.00^{bC}	6.54± 0.00^{aC}	6.62±0.00 ^{bA}	6.57± 0.00^{aB}

Esphericity (%)	67.35± 0.00^{aA}	64.11± 0.00^{aC}	64.94±0.00 ^{aB}	65.21±0.00 ^{bC}	65.73± 0.00^{aA}	64.46± 0.00^{bC}
Circularity (%)	80.63±0.00 ^{aA}	70.29±0.00 ^{bC}	71.63±0.00 ^{aB}	71.00± 0.00^{bB}	72.68±0.00 ^{aA}	70.34±0.00 ^{bC}
Surface área (mm²)	113.82± 0.00^{bB}	114.96± 0.00^{bA}	111.55± 0.00^{bC}	116.61±0.00 ^{aC}	119.39±0.00 ^{aA}	116.69± 0.00^{aB}
Volume (mm³)	115.52± 0.00^{bB}	115.88±0.00 ^{aA}	111.93±0.00 ^{bC}	117.96±0.00 ^{aB}	121.52±0.00 ^{aA}	117.80± 0.00^{aC}

[1] Means ± standard deviation followed by different lower case letters horizontally show statistical differences using the Tukey test ($p < 0.05$), different upper case letters vertically horizontally show statistical differences using the t-Student ($p < 0.05$).

Table S2. Physico-chemical and microbiological analysis of Coffea arabica grains from the Alta Mogiana region subjected to different drying methods during different harvest periods[1].

Parameters	Alto Mogiana					
	Solar Dryer			Coffeedryer		
	Begin	Middle	End	Begin	Middle	End
Weight of 100 green coffee beans(g)	9.18±0.08^{bB}	9.82±0.13^{bA}	9.86±0.07^{bA}	11.46±0.14^{aA}	11.38±0.08^{aA}	11.34±0.08^{aA}
Aw	0.52±0.00^{aC}	0.51±0.00^{aB}	0.53±0.00^{aA}	0.46±0.00^{bB}	0.46±0.00^{aA}	0.47±0.00^{bA}
L*	49.19±0.51^{bA}	49.61±0.40^{aA}	48.10±0.16^{bB}	49.93±0.35^{aA}	49.20±0.28^{aB}	49.67±0.17^{aA}
ºHUE	83.45±0.44^{aA}	83.77±0.34^{aA}	83.91±0.24^{aA}	85.45±0.75^{aA}	83.55±0.21^{aA}	83.65±0.43^{aA}
C*	16.49±0.19^{bA}	16.52±0.37^{bA}	16.04±0.15^{bA}	17.20±0.12^{aA}	17.28±0.41^{aA}	17.63±0.36^{aA}
Moisture(%)	7,73±0.05^{bB}	7.76±0.09^{bB}	8,86±0.11^{aA}	8,21±0.14^{aB}	9.01±0.38^{aA}	8.11±0.05^{bB}
Lipids (%)	3.37±0.50^{aA}	2.93±0.46 ^{aA}	3.28±0.39 ^{aA}	3.10±0.41 ^{aA}	3.41±0.60 ^{aA}	3.33±0.52 ^{aA}
β-Carotene bleaching (%protection)	28.43±4.17^{bA}	29.72±5.64^{bA}	26.81±2.25^{bA}	51.14±4.64^{aA}	45.05±6.00^{aA}	47.71±4.80^{aA}
Phenolics compounds (g EAG. 100g^{-1})	2.16±0.07^{bB}	2.22±0.15^{bA}	2.21±0.09^{bA}	3.53±0.33^{aA}	3.00±0.05^{aB}	3.20±0.13^{aB}
pH	6.31±0.01^{bA}	6.27±0.01^{bB}	6.22±0.01^{bC}	6.34±0.03^{aA}	6.30±0.01^{aB}	6.32±0.00^{aB}
ATT (%)	196.66±5.77^{aB}	236.66±5.77^{aA}	245.00±0.00^{aA}	180.00±10.00^{bB}	206.66±5.77^{bA}	190.00±0.00^{bB}
SS (%)	31.66±0.58^{bB}	33.33±1.15^{aB}	36.33±0.58^{aA}	36.67±1.53^{aA}	34.00±3.00^{aA}	34.66±0.58^{aA}
Ash (%)	5.04±0.12^{aA}	5.05±0.09^{bA}	5.06±0.10^{bA}	5.25±0.02^{aA}	5.34±0.23^{aA}	5.34±0.17^{aA}
Protein (%)	0.18±0.01^{aA}	0.18±0.01^{aA}	0.18±0.01^{aA}	0.18±0.00^{aA}	0.18±0.01^{aA}	0.17±0.01^{aA}
Total Carbohydrate (%)	83.66±0.40^{aA}	84.06±0.47^{aA}	82.60±0.37^{aB}	83.24±0.52^{bA}	82.04±0.79^{aB}	83.03±0.34^{aA}
Hygienic-sanitary quality (log. UFC.g^{-1})	5.02±0.14^{aA}	3.99±0.00^{bC}	4.87±0.14^{aB}	4.33±0.03^{bB}	4.61±0.02^{aA}	0.00±0.00^{bC}
Length (mm)	8.49±0.00^{bA}	8.42±0.00^{bB}	8.29±0.00^{aC}	9.30±0.00^{aA}	9.23±0.00^{aB}	6.56±0.00^{bC}
Width (mm)	6.36±0.00^{bA}	6.23±0.00^{bB}	6.24±0.00^{aC}	6.69±0.00^{aA}	6.58±0.00^{aB}	3.27±0.00^{bC}
Thickness (mm)	3.51±0.00^{bA}	3.50±0.00^{bB}	3.46±0.00^{bC}	4.10±0.00^{aB}	4.11±0.00^{aA}	3.67±0.00^{aC}
Geometric diameter (mm)	5.73±0.00^{bA}	5.68±0.00^{bB}	5.63±0.00^{bC}	6.27±0.00^{aA}	6.23±0.00^{aB}	6.07±0.00^{aC}
Arithmetic diameter (mm)	6.12±0.00^{bA}	6.05±0.00^{bB}	6.00±0.00^{bC}	6.70±0.00^{aA}	6.64±0.00^{aB}	6.51±0.00^{aC}
Esphericity (%)	67.68±0.00^{aB}	67.60±0.00^{aC}	68.36±0.00^{aA}	67.48±0.00^{bB}	67.50±0.00^{bA}	65.36±0.00^{bC}
Circularity (%)	75.12±0.00^{aB}	74.33±0.00^{aC}	75.62±0.00^{aA}	72.14±0.00^{bA}	71.43±0.00^{bB}	70.76±0.00^{bC}
Surface área (mm²)	103.40±0.00^{bA}	101.37±0.00^{bB}	99.68±0.00^{bC}	125.06±0.00^{aA}	123.24±0.00^{aB}	115.71±0.00^{aC}
Volume (mm³)	98.99±0.00^{bA}	96.81±0.00^{bB}	93.66±0.00^{bC}	129.40±0.00^{aA}	127.85±0.00^{aB}	117.07±0.00^{aC}

[1] Means ± standard deviation followed by different lower-case letters horizontally show statistical differences using the Tukey test ($p<0.05$), different upper case letters vertically horizontally show statistical differences using the t-Student ($p<0.05$).Table S3. Notes on the sensory evaluation of coffee

beverage from genetic varieties of arabica coffee from Campo das vertentes subjected to different drying methods.

Table S2. Cont.

Parameters	Solar + Mechanical Dryer			Coffeedryer		
	**Begin**	**Middle**	**End**	**Begin**	**Middle**	**End**
Fragrance/Aroma	7.00bA	7.00bA	7.00bA	7.50aA	7.33aA	7.16aA
Uniformity	10.00aA	10.00aA	10.00aA	10.00aA	10.00aA	10.00aA
Clean Cup	10.00aA	10.00aA	10.00aA	10.00aA	10.00aA	10.00aA
Sweetness	10.00aA	10.00aA	10.00aA	10.00aA	10.00aA	10.00aA
Flavor	7.00bA	7.00bA	7.00bA	7.50aA	7.33aA	7.16aA
Acidity	7.00bA	7.00aA	7.00aA	7.33aA	7.00aB	7.00aB
Body	7.00bA	7.00aA	7.00aA	7.33aA	7.00aB	7.00aB
Aftertaste	7.00aA	7.00aA	7.00aA	7.00aA	7.00aA	7.00aA
Balance	7.00aA	7.00aA	7.00aA	7.00aA	7.00aA	7.00aA
Overall	7.00aA	7.00aA	7.00aA	7.00aA	7.00aA	7.00aA
Final score	79.00bA	79.00bA	79.00aA	80.93aA	80.00aB	79.33aB

[1] Means followed by different lower-case letters horizontally show statistical differences using the Tukey test ($p<0.05$), different upper-case letters vertically horizontally show statistical differences using the t-Student ($p<0.05$).Table S4. Notes on the sensory evaluation of coffee beverage from genetic varieties of arabica coffee from Alta Mogiana subjected to different drying methods[1].

Table S2. Cont.

Parameters	Solar Dryer			Coffeedryer		
	**Begin**	**Middle**	**End**	**Begin**	**Middle**	**End**
Fragrance/Aroma	7.00aA	7.16bA	7.00bA	7.33aA	7.33aA	7.50aA
Uniformity	10.00aA	10.00aA	10.00aA	10.00aA	10.00aA	10.00aA
Clean Cup	10.00aA	10.00aA	10.00aA	10.00aA	10.00aA	10.00aA
Sweetness	10.00aA	10.00aA	10.00aA	10.00aA	10.00aA	10.00aA
Flavor	7.00aA	7.16aA	7.00bA	7.33aA	7.33aA	7.50aA
Acidity	7.00aA	7.00aA	7.00aA	7.00aA	7.00aA	7.00aA
Body	7.00aA	7.00aA	7.00aA	7.00aA	7.00aA	7.00aA
Aftertaste	7.00aA	7.00aA	7.00aA	7.00aA	7.00aA	7.00aA
Balance	7.00aA	7.00aA	7.00aA	7.00aA	7.00aA	7.00aA
Overall	7.00aA	7.00aA	7.00aA	7.00aA	7.00aA	7.00aA
Final score	79.00bA	79.00bA	79.00bA	80.00aA	80.00aA	80.00aA

[1] Means followed by different lower case letters horizontally show statistical differences using the Tukey test ($p<0.05$), different upper case letters vertically horizontally show statistical differences using the t-Student ($p<0.05$).
